# Ugi post-condensation copper-triggered oxidative cascade towards pyrazoles

**DOI:** 10.3762/bjoc.7.153

**Published:** 2011-09-21

**Authors:** Aurélie Dos Santos, Laurent El Kaim, Laurence Grimaud, Caroline Ronsseray

**Affiliations:** 1 Laboratoire Chimie et Procédés, Laboratoire Chimie et procédés, UMR 7652-DCSO-CNRS- ENSTA-École Polytechnique, École Nationale Supérieure de Techniques Avancées, 32 Bd Victor, Paris 75015, France

**Keywords:** aerobic oxidation, copper(II), [3 + 2] cycloaddition, hydrazone, isocyanide, pyrazolidinone

## Abstract

Pyrazolidinones were prepared in a two-step sequence starting from α-hydrazonocarboxylic acids. After a four-component Ugi coupling, the resulting hydrazone was engaged in a copper triggered [3 + 2] cycloaddition/aerobic oxidation cascade.

## Introduction

In the last twenty years, the Ugi reaction coupled with its various post-condensations towards heterocyclic libraries has established the success of isocyanide-based multicomponent reactions [1–7]. Chemists in both academia and industry have taken advantage of the functional group tolerance of the Ugi coupling to apply to these adducts the various cyclizations offered by the chemists toolkit. We became involved in the Ugi-post-condensation field through our initial interest in radical processes. We found that, compared with classical cycloadditions, cyclocondensations and organometalic couplings, there was no existing description of radical processes on such adducts. Thus, we decided to undertake various studies using xanthate transfer [8–10], Mn(III) or copper(II) triggered oxidative couplings [11–12].

**Scheme 1:**
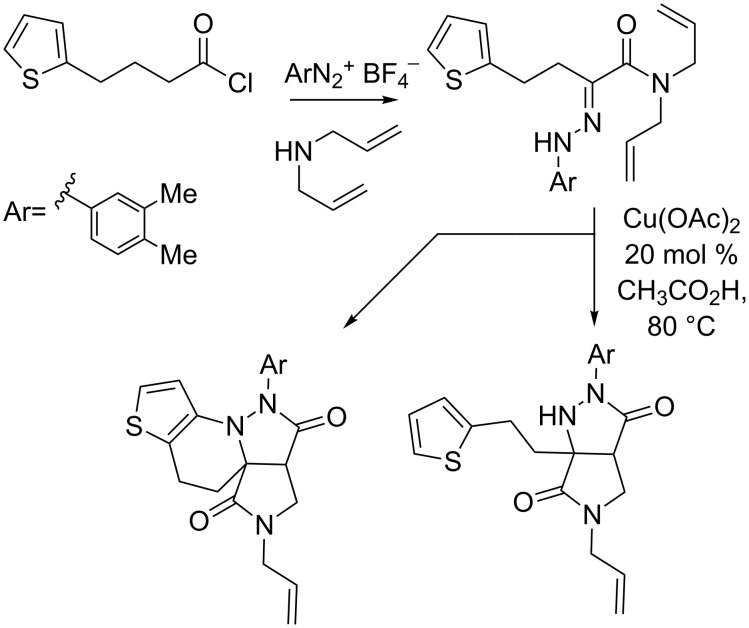
Copper-catalyzed oxidative cyclization of alkenyl hydrazone.

We recently reported a new synthesis of fused pyrazolidinone under oxidative conditions from simple hydrazone derivatives (Scheme 1) [13]. The cascade features a [3 + 2] cycloaddition coupled with an aerobic oxidation of the resulting pyrazolidine. A further oxidative coupling may be observed according to the substitution pattern of the starting acyl chloride. Considering our interest in IMCR, we envisioned that a similar cascade could be performed on a properly functionalized Ugi adduct allowing us to reach a new 4-component access to pyrazole derivatives. The present letter summarizes our efforts in this direction.

## Results and Discussion

Among the possible Ugi pathways to introduce an alkene moiety that is prone to undergo an intramolecular [3 + 2] cycloaddition with a hydrazone, we selected the Ugi coupling between α-hydrazonocarboxylic acids and allylamine as the most straightforward path. There are several reports on the use of hydrazones in Ugi reactions [14–23], however, to the best of our knowledge, there is no report involving α-hydrazonocarboxylic acids.

**Scheme 2:**
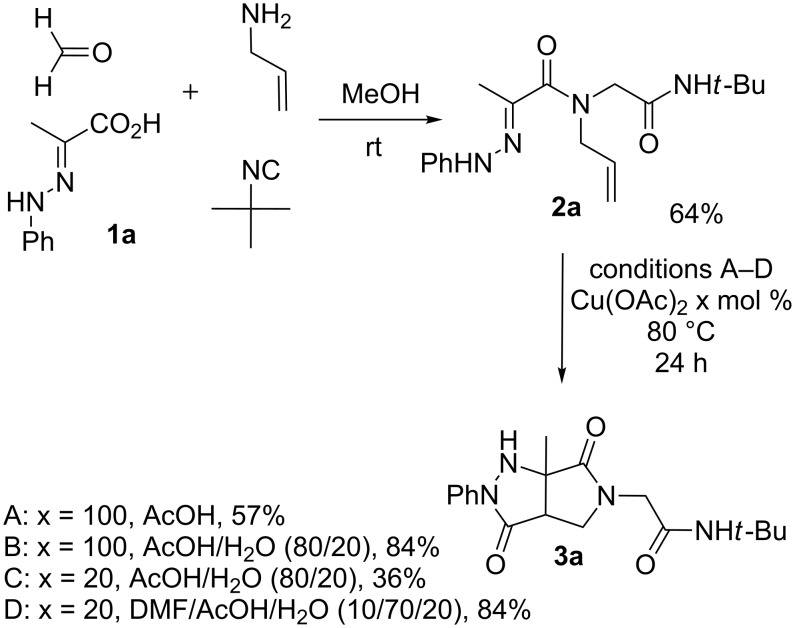
Pyrazolidinone **3a** from Ugi adduct **2a**.

Hydrazone **1a** was prepared through condensation of pyruvic acid with phenylhydrazine. Adding **1a** to aqueous formaldehyde, allylamine and *tert*-butylisocyanide in MeOH under standard Ugi conditions, led to the formation of the amide **2a** in 64% isolated yield. The compatibility of the hydrazone with this coupling is certainly due to the higher electrophilicity of the intermediate iminium. The latter traps the isocyanide before any interaction with the hydrazone. The first attempted oxidative cyclization of **2a** was made with one equivalent of copper acetate in acetic acid as solvent and gave the expected pyrazolidinone **3a** in a 57% isolated yield (Scheme 2, condition A). Based on our previous study, the yield was improved to reach 84% with a mixture of acetic acid and water (80/20). A combination of DMF, acetic acid and water allowed us to optimize this reaction working with a reduced 20 mol % of copper (84% isolated yield, Scheme 2, condition D). The reaction was performed at 80 °C, overnight, and under argon. We believe that under these conditions a slow uptake of oxygen helps to control the selective oxidation process. Reactions performed under air were faster but led to intractable mixtures.

**Scheme 3:**
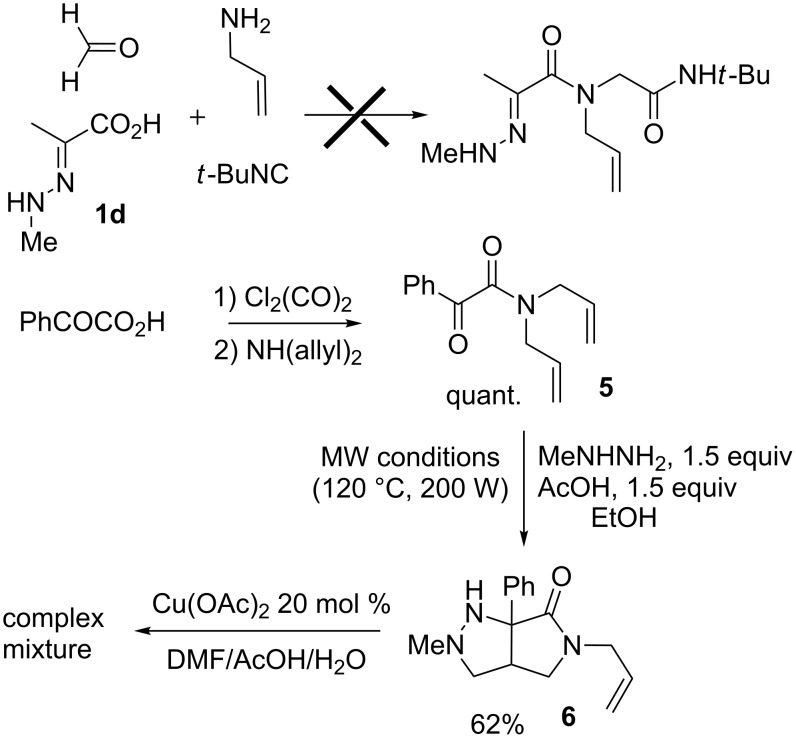
Attempted reactions of N-methyl hydrazones.

Analogous hydrazones prepared from pyruvic acid and benzoylformic acid with hydrazine derivatives were tested in this Ugi/oxidative cyclization sequence under these optimized conditions. Results are reported in Table 1. Surprisingly, the reaction appears to be only efficient with Ugi adducts prepared with formaldehyde as the carbonyl component (Table 1, entries 1–5). With other aldehydes and ketones, even if the Ugi reaction was performed easily, the following cyclization failed to give the expected pyrazolidinones and resulted in complex mixture formation. Intermediate Ugi adduct **3g** (Table 1, entry 6) only resulted in a small amount of ring-opened product **4g**. The reaction is also limited to *N*-aryl hydrazones due to the lower efficiency of the Ugi reaction with *N*-alkyl hydrazones: An attempt of Ugi coupling with hydrazone **1d**, formaldehyde, allylamine and *tert*-butylisocyanide failed to give any isolable adduct (Scheme 3). This may be explained by an enhanced nucleophilicity of the *N*-monoalkyl hydrazone leading to a competition between the hydrazone and the amine component in the Ugi steps.

**Table 1:** Cycloaddition/oxidation cascade from Ugi hydrazone adducts.

Entry	Ugi Product	Cycloadduct

1	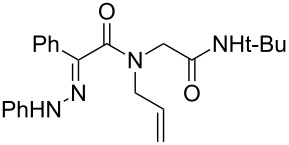 **2b**, 79%	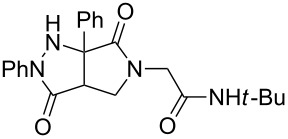 **3b**, 68%
2	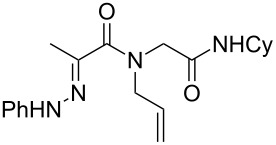 **2c**, 78%	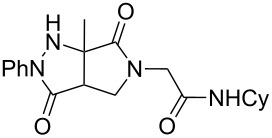 **3c**, 76%
3	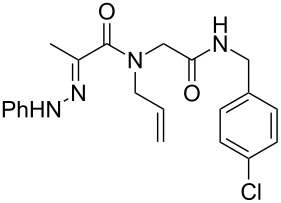 **2d**, 71%	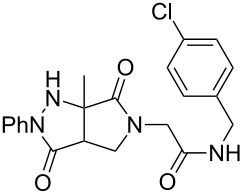 **3d**, 90%
4	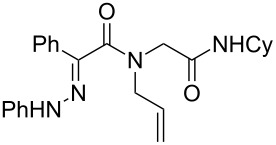 **2e**, 94%	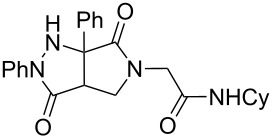 **3e**, 72%
5	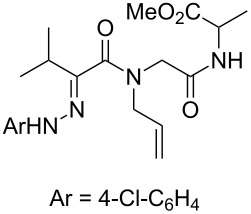 **2f**, 37%	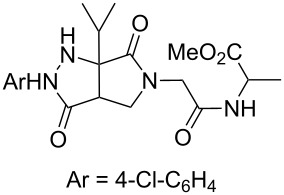 **3f**, 49%
6	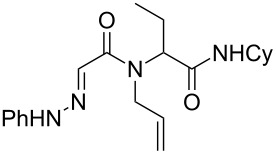 **3g**, 58%	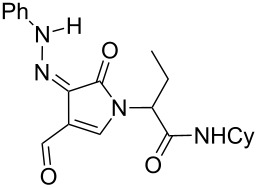 **4g**, 12%
7	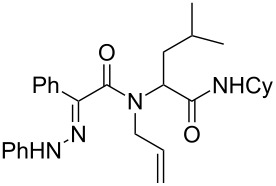 **3h**, 52%	–
8	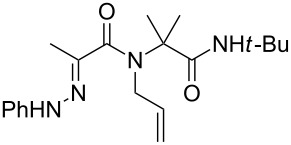 **3i**, 50%	–

In order to gain further insight into the reactivity of *N*-alkyl derivatives, we decided to synthesize an initial hydrazone by a more conventional route. Benzoylformic acid was converted into its *N*-diallyl amide derivative. The latter failed to produce a hydrazone with methylhydrazine under standard conditions (EtOH, toluene, rt to reflux, with or without added acetic acid). However, we were able to trigger the addition under microwave conditions (in EtOH with 1.5 equiv of AcOH). The expected hydrazone was still not synthesized, however, the cycloadduct **6** was obtained, probably through a [3 + 2] cycloaddition triggered by acetic acid. The attempted oxidation of **6** with copper acetate in DMF/AcOH/H_2_O gave only complex mixtures.

**Scheme 4:**
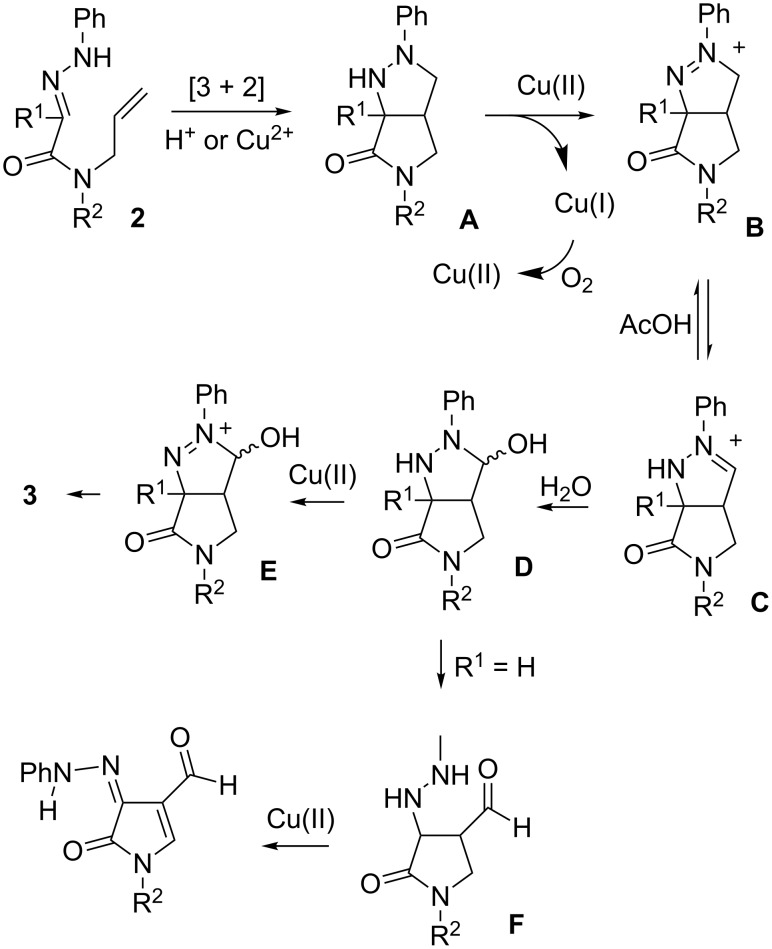
Proposed mechanism.

The oxidation sequence may be explained by the mechanism depicted in Scheme 4. The process starts with a [3 + 2] cycloaddition triggered either by copper acetate or acetic acid [24–29]. The resulting pyrazoline **A** may be oxidized by copper(II) salts forming intermediate **D** after addition of water [30–31]. Two alternative paths may then be observed from **D**: Ring-opening leading to azo or hydrazono derivatives such as **4g**, further oxidation without ring-opening giving the fused pyrazolidinone **3**.

## Conclusion

In conclusion, we have disclosed a new Ugi coupling with α-hydrazonocarboxylic acids. These Ugi adducts have been used in an Ugi post-condensation involving a [3 + 2] cycloaddition followed by an oxidative cascade. Among potential Ugi post-condensations, radical and oxidative processes represent a very promising route towards the formation of complex scaffolds. We are currently exploring the reactivity of the *N*-aryl Ugi–Smiles adducts using similar strategies.

## Experimental

**Typical procedure for the first step: (*E*)-*N*-allyl-*N*-(2-(*tert*-butylamino)-2-oxoethyl)-2-(2-phenylhydrazono)propanamide (2a):** To a solution of formaldehyde (210 μL, 2.8 mmol) in methanol (1 M) were added successively allylamine (210 μL, 2.8 mmol), 2-(2-phenylhydrazono)propanoic acid (500 mg, 2.8 mmol), and *tert*-butylisocyanide (230 mg, 2.8 mmol). The resulting mixture was stirred at 40 °C until completion of the reaction (TLC). The solvent was removed under reduced pressure. The product was isolated by flash chromatography on silica gel (PE/Et_2_O) with a yield of 64%. ^1^H NMR (CDCl_3_, 400 MHz) δ 7.52 (br s, 1H), 7.29 (dd, *J =* 7.8, 7.3 Hz, 2H), 7.09 (d, *J =* 7.8 Hz, 2H), 6.95 (t, *J =* 7.3 Hz, 1H), 6.16 (br s, 1H), 5.96–5.88 (m, 1H), 5.28–5.23 (m, 2H), 4.34–4.00 (m, 4H), 2.14 (s, 3H), 1.35 (s, 9H); ^13^C NMR (CDCl_3_, 100.6 MHz) δ 168.7, 168.6, 143.9, 136.9, 132.8, 129.8, 122.0, 119.0, 114.0, 53.9, 51.7, 51.3, 29.1, 12.6.

**Typical procedure for the oxidative cyclization: *N*-*tert*-butyl-2-(6a-methyl-3,6-dioxo-2-phenylhexahydropyrrolo[3,4-c]pyrazol-5(1*H*)-yl)acetamide (3a):** To a solution of hydrazone **2a** (100 mg, 0.3 mmol) in a 10/70/20 DMF/CH_3_COOH/H_2_O mixture (0.06 M) was added Cu(OAc)_2_ (20 mol %). The resulting mixture was heated at 80 °C under argon. The pH was adjusted to 6 with an aqueous sodium hydrogencarbonate solution, and the aqueous phase was extracted with AcOEt. Then the organic layers were washed ten times with water, dried over anhydrous MgSO_4_, filtered and concentrated in vacuo. The product was isolated by flash chromatography on silica gel (PE/Et_2_O with 1% of TEA) with a yield of 84%. ^1^H NMR (CDCl_3_, 400 MHz) δ 7.83 (d, *J =* 8.3 Hz, 2H), 7.37 (dd, *J =* 8.3, 7.3 Hz, 2H), 7.17 (t, *J =* 7.3 Hz, 1H), 5.52 (br s, 1H, NH), 4.89 (br s, 1H), 3.93 (d, *J =* 16.2 Hz, 1H), 3.85 (dd, *J =* 10.3, 6.3 Hz, 1H), 3.80 (d, *J =* 16.2 Hz, 1H), 3.76 (d, *J =* 10.3 Hz, 1H), 3.22 (d, *J =* 6.3 Hz, 1H), 1.63 (s, 3H), 1.25 (s, 9H); ^13^C NMR (CDCl_3_, 100.6 MHz) δ 174.6, 169.8, 166.3, 138.1, 129.2, 125.8, 119.5, 63.7, 52.0, 48.0, 47.6, 29.0, 18.9.

## Supporting Information

File 1 Experimental procedures with characterization data for all new compounds.
