# Supplementary material for: Ugi post-condensation copper-triggered oxidative cascade towards pyrazoles
Source: Beilstein J Org Chem. 2011 Sep 21;7:1310–4. doi: 10.3762/bjoc.7.153 (PMC3201043; doi:10.3762/bjoc.7.153)
Supplement: File 1 — Experimental procedures with characterization data for all new compounds. [file Beilstein_J_Org_Chem-07-1310-s001.pdf]

Supporting Information

for

Ugi post-condensation copper-triggered oxidative cascade towards pyrazoles

Aurélie Dos Santos, Laurent El Kaim*, Laurence Grimaud* and Caroline Ronsseray

Address: Laboratoire Chimie et Procédés, Laboratoire Chimie et procédés, UMR 7652-DCSO-CNRS- ENSTA-École Polytechnique, École Nationale Supérieure de Techniques Avancées, 32 Bd Victor, Paris 75015, France

Email: Laurent El Kaim - laurent.elkaim@ensta.fr; Laurence Grimaud - laurence.grimaud@ensta.fr

* Corresponding author

Table of contents:

I. **General procedures** **S2**

II. **General Procedure for Syntheses** **S3**

III. **Spectral data for products** **S4–S20**

I. General procedures

^1H NMR spectra were recorded on a Bruker Avance 400 spectrometer, using $CDCl_3$ solvent as reference and/or internal deuterium lock. ^{13}C NMR spectra were recorded on a Bruker Avance 400 (100.6 MHz) spectrometer. Two-dimensional NMR spectroscopy [^1H-^1H COSY spectra, ^1H-^{13}C COSY spectra (HSQC) and long-range ^1H-^{13}C COSY spectra (HMBC)], were carried out to determine the correlation between ^1H and ^{13}C. The chemical shifts for all NMR spectra are expressed in parts per million up to the high frequency of the TMS reference. Coupling constants (*J*) are quoted in Hz and are recorded to the nearest 0.1 Hz.

The IR spectra were obtained on a Bruker IFS 66 or a Perkin-Elmer FT 1600 spectrometer. Low resolution mass spectral analysis (EI and CI) were recorded on a Hewlett-Packard HP5989 mass spectrometer by either direct injection or GC/MS coupling with a Hewlett-Packard HP5890 chromatograph. High resolution (HR) mass spectra were performed on a JEOL JMS-GCmate II, GC/MS system spectrometer. TLC was carried out on precoated plates of silica gel $60F_{254}$.

II. General Procedure for Syntheses

General Procedure A: preparation of Ugi adducts:

To a solution of aldehyde (1 equiv) in methanol (1 M) were added successively allylamine (1 equiv), hydrazono acetic acid (1 equiv) and isocyanide (1 equiv). The resulting mixture was stirred at 40 °C until completion of the reaction (TLC). The solvent was removed under reduced pressure. The product was isolated by flash chromatography on silica gel (PE/Et_2O).

General Procedure B: preparation of pyrrazolidinone:

To a solution of hydrazone **1** (1 equiv) in a 10/70/20 DMF/CH_3COOH/H_2O mixture (0.06 M) was added $Cu(OAc)_2$ (20 mol %). The resulting mixture was heated at 80 °C under argon. The pH was adjusted to 6 with an aqueous sodium hydrogencarbonate solution, the aqueous phase was extracted with AcOEt. Then the organic layers were washed ten times with water, dried over anhydrous $MgSO_4$, filtered and concentrated in vacuo. The product was isolated by flash chromatography on silica gel (PE/Et_2O with 1 % of TEA).

III. Spectral data for products from Table 1

(*E*)-*N*-allyl-*N*-(2-(*tert*-butylamino)-2-oxoethyl)-2-(2-phenylhydrazono)propanamide (2a)

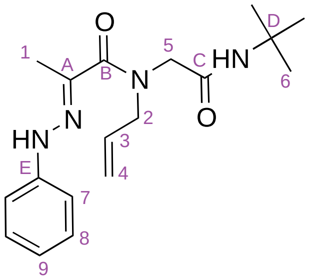

$C_{18}H_{26}N_4O_2$
Mol wt = 330.21 g·mol^{-1}

Compound 2a was prepared following the general procedure A applied to 2-(2-phenylhydrazono)-propanoic acid (500 mg, 2.8 mmol), formaldehyde (210 µL, 2.8 mmol), *tert*-butylisocyanide (230 mg, 2.8 mmol).

Yield: 64% (600 mg)

R_f : 0.2 (3:7 P.E./Et_2O)

White solid.

Mp: 137–138 °C

^{1}H NMR ($CDCl_3$, 400 MHz) δ 7.52 (br s, 1H, NH) 7.29 (dd, J = 7.8, 7.3 Hz, 2H, H_8), 7.09 (d, J = 7.8 Hz, 2H, H_7), 6.95 (t, J = 7.3 Hz, 1H, H_9), 6.16 (br s, 1H, NH), 5.96–5.88 (m, 1H, H_3), 5.28–5.23 (m, 2H, H_4), 4.34–4.00 (m, 4H, H_2, H_5), 2.14 (s, 3H, H_1), 1.35 (s, 9H, H_6).

^{13}C NMR ($CDCl_3$, 100.6 MHz) δ 168.7 (C_C), 168.6 (C_B), 143.9 (C_E), 136.9 (C_A), 132.8 (C_3), 129.8 (C_8), 122.0 (C_9), 119.0 (C_4), 114.0 (C_7), 53.9 (C_2), 51.7 (C_5), 51.3 (C_D), 29.1 (C_6), 12.6 (C_1).

IR (ν, cm^{-1}): 1684, 1653, 1558, 1541, 1508, 1457.

HRMS: calculated for $C_{18}H_{26}N_4O_2$ 330.2056, found 330.2064.

(*E*)-*N*-allyl-*N*-(2-(*tert*-butylamino)-2-oxoethyl)-2-phenyl-2-(2-phenylhydrazono) acetamide (2b)

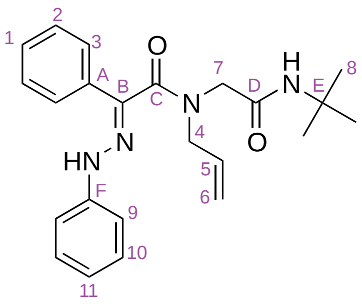

$C_{23}H_{28}N_4O_2$
Mol wt = 392.49 g·mol^{-1}

Compound 2b was prepared following the general procedure A applied to 2-phenyl-2-(2-phenylhydrazono)acetic acid (500 mg, 2.1 mmol), formaldehyde (160 µL, 2.1 mmol), *tert*-butylisocyanide (175 mg, 2.1 mmol).

Yield: 79% (868 mg)

R_f: 0.5 (3:7 PE/Et_2O)

White solid.

Mp: 169–170 °C

^{1}H NMR ($CDCl_3$, 400 MHz) δ 10.29 (br s, 1H, NH), 7.73 (d, *J* = 7.3 Hz, 2H, H_3), 7.41–7.37 (m, 4H, H_2, H_9), 7.33–7.29 (m, 3H, H_1, H_{10}), 6.90 (t, *J* = 7.1 Hz, 1H, H_{11}), 5.68–5.58 (m, 1H, H_5), 5.48 (br s, 1H, NH), 5.16–5.13 (m, 2H, H_6), 4.14 (m, 2H, H_7), 3.94 (d, *J* = 6.1 Hz, 2H, H_4), 1.44 (s, 9H, H_8).

^{13}C NMR ($CDCl_3$, 100.6 MHz) δ 167.6 (C_D), 167.5 (C_C), 145.5 (C_F), 136.7 (C_B), 134.7 (C_A), 132.9 (C_5), 129.5 (C_{10}), 129.0 (C_2), 128.5 (C_1), 125.5 (C_3), 120.5 (C_{11}), 119.7 (C_6), 113.5 (C_9), 52.7 (C_E), 52.1 (C_4), 47.6 (C_7), 29.3 (C_8).

IR (ν, cm^{-1}): 1653, 1558, 1541, 1508, 1457.

HRMS: calculated for $C_{23}H_{28}N_4O_2$ 392.2212, found 392.2217.

(E)-N-allyl-N-(2-(cyclohexylamino)-2-oxoethyl)-2-(2-phenylhydrazono) propanamide (2c)

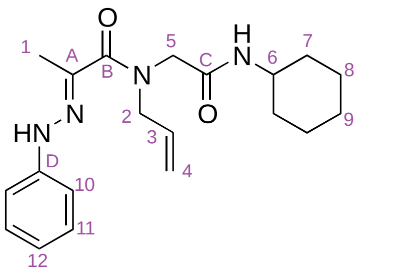

$C_{20}H_{28}N_4O_2$
Mol wt = 356.46 g·mol^{-1}

Compound **2c** was prepared following the general procedure A applied to 2-(2-phenylhydrazono) propanoic acid (500 mg, 2.8 mmol), formaldehyde (210 µL, 2.8 mmol), cyclo-hexylisocyanide (403 µl, 2.8 mmol).

Yield: 78% (780 mg)

R_f: 0.1 (3:7 PE/Et_2O)

Brown solid.

Mp: 95–96 °C

^{1}H NMR ($CDCl_3$, 400 MHz) δ 7.43 (br s, 1H, NH), 7.29–7.25 (m, 2H, H_{11}), 7.05 (d, J = 7.3 Hz, 2H, H_{10}), 6.94 (t, J = 8.5 Hz, 1H, H_{12}), 6.31–6.11 (m, 1H, NH), 5.92–5.85 (m, 1H, H_3), 5.27–5.21 (m, 2H, H_4), 4.36–4.18 (m, 2H, H_5), 4.15–4.03 (m, 2H, H_2), 3.85–3.76 (m, 1H, H_6), 2.13 (s, 3H, H_1), 1.89–1.86 (m, 2H, H_7), 1.68–1.63 (m, 2H, H_8), 1.61–1.56 (m, 2H, H_9), 1.36–1.30 (m, 2H, H_8), 1.19–1.10 (m, 2H, H_7).

^{13}C NMR ($CDCl_3$, 100.6 MHz) δ 168.1 (C_B, C_C), 143.5 (C_D), 136.2 (C_A), 131.7 (C_3), 129.2 (C_{11}), 121.5 (C_{12}), 118.7 (C_4), 113.6 (C_{10}), 53.3 (C_2), 50.7 (C_5), 48.3 (C_6), 32.8 (C_7), 25.4 (C_9), 24.6 (C_8), 12.1 (C_1).

IR (v, cm^{-1}): 1653, 1597, 1541, 1496, 1454, 1424, 1244.

HRMS: calculated for $C_{20}H_{28}N_4O_2$ 356.2212, found 356.2215

(*E*)-*N*-allyl-*N*-(2-(4-chlorobenzylamino)-2-oxoethyl)-2-(2-phenylhydrazono) propanamide (2d)

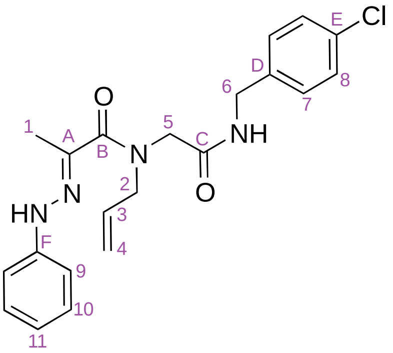

$C_{21}H_{23}ClN_4O_2$
Mol wt = 398.89 g·mol^{-1}

Compound **2d** was prepared following the general procedure A applied to 2-(2-phenylhydrazono) propanoic acid (500 mg, 2.8 mmol), formaldehyde (210 µL, 2.8 mmol), *p*-chlorobenzylisocyanide (420 mg, 2.8 mmol).

Yield: 71% (800 mg)

R_f : 0.1 (3:7 PE/Et_2O)

Yellow solid.

Mp: 96–97 °C

^{1}H NMR ($CDCl_3$, 400 MHz) δ 7.51 (br s, 1H, NH) 7.29–7.24 (m, 4H, H_8, H_{10}), 7.19–7.17 (m, 2H, H_7), 7.03 (d, J = 7.3 Hz, 2H, H_9), 6.96 (t, J = 7.3 Hz, 1H, H_{11}), 6.91 (br s, 1H, NH), 5.94–5.87 (m, 1H, H_3), 5.32–5.25 (m, 2H, H_4), 4.43 (d, J = 6.0 Hz, 2H, H_6), 4.28–4.16 (m, 4H, H_2, H_5), 2.09 (s, 3H, H_1).

^{13}C NMR ($CDCl_3$, 100.6 MHz) δ 169.6 (C_C), 168.7 (C_B), 143.8 (C_F), 136.9 (C_D), 136.8 (C_A), 133.6 (C_E), 132.9 (C_3), 129.8 (C_8), 129.4 (C_{10}), 129.2 (C_7), 122.1 (C_{11}), 119.2 (C_4), 114.4 (C_9), 53.7 (C_2), 50.9 (C_5), 43.1 (C_6), 12.5 (C_1).

IR (ν, cm^{-1}): 1601, 1541, 1492, 1458, 1242.

HRMS: calculated for $C_{21}H_{23}ClN_4O_2$ 398.1510, found 398.1516.

(*E*)-*N*-allyl-*N*-(2-(cyclohexylamino)-2-oxoethyl)-2-phenyl-2-(2-phenylhydrazono) acetamide (2e)

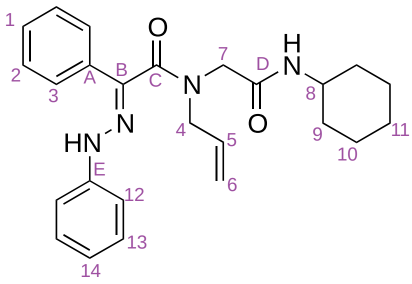

$C_{25}H_{30}N_4O_2$
Mol wt = 418.53 g·mol^{-1}

Compound **2e** was prepared following the general procedure A applied to 2-phenyl-2-(2-phenylhydrazono)acetic acid (673 mg, 2.8 mmol), formaldehyde (210 µL, 2.8 mmol), cyclo-hexylisocyanide (403 µL, 2.8 mmol).

Yield: 94% (1.1 g)

Rf : 0.3 (3:7 PE/Et_2O)

White solid.

Mp: 184–185 °C

^{1}H NMR ($CDCl_3$, 400 MHz) δ 9.99 (br s, 1H, NH), 7.63 (d, *J* = 7.6 Hz, 2H, H_3), 7.31–7.27 (m, 3H, H_1, H_{12}), 7.24–7.19 (m, 4H, H_2, H_{13}), 6.81 (t, *J* = 7.3 Hz, 1H, H_{14}), 5.64 (br s, 1H, NH), 5.59–5.49 (m, 1H, H_5), 5.07–5.03 (m, 2H, H_6), 4.10 (s, 2H, H_7), 3.86 (d, *J* = 5.8 Hz, 2H, H_4), 3.85–3.76 (m, 1H, H_8), 1.91–1.87 (m, 2H, H_9), 1.65–1.62 (m, 2H, H_{10}), 1.57–1.52 (m, 2H, H_{11}), 1.33–1.27 (m, 2H, H_{10}), 1.13–1.06 (m, 2H, H_9).

^{13}C NMR ($CDCl_3$, 100.6 MHz) δ 167.5 (C_D), 167.3 (C_C), 145.3 (C_E), 136.7 (C_B), 134.7 (C_A), 132.9 (C_5), 129.5 (C_2), 129.0 (C_{13}), 128.6 (C_1), 125.5 (C_3), 120.6 (C_{14}), 119.9 (C_6), 113.6 (C_{12}), 52.2 (C_4), 49.4 (C_8), 47.2 (C_7), 33.4 (C_9), 26.0 (C_{11}), 25.3 (C_{10}).

IR (v, cm^{-1}): 1653, 1624, 1558, 1541, 1507, 1457.

HRMS: calculated for $C_{25}H_{30}N_4O_2$ 418.2369, found 418.2375.

(*E*)-methyl 2-(2-(*N*-allyl-2-(2-(4-chlorophenyl)hydrazono)-3-methylbutanamido) acetamido)propanoate (2f)

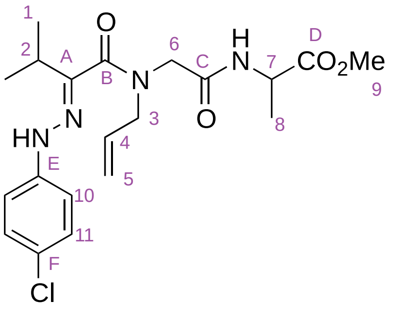

$C_{20}H_{27}ClN_4O_4$
Mol wt = 422.91 g·mol^{-1}

Compound **2f** was prepared following the general procedure A applied to 2-(2-(4-chlorophenyl)hydrazono)-3-methylbutanoic acid (78 mg, 0.32 mmol), formaldehyde (25 µL, 0.32 mmol), methyl 2-isocyanopropanoate (37 mg, 0.32 mmol).

Yield: 37% (50 mg)

R_f: 0.3 (100% Et_2O)

Yellow oil.

^{1}H NMR ($CDCl_3$, 400 MHz) δ 7.55 (br s, 1H, NH), 7.15 (d, J = 8.6 Hz, 2H, H_{11}), 7.09 (d, J = 8.6 Hz, 2H, H_{10}), 6.57 (br s, 1H, NH), 5.70 (ddt, J = 16.9, 10.8, 5.6 Hz, 1H, H_4), 5.23 (d, J = 10.8 Hz, 1H, H_5), 5.18 (d, J = 16.9 Hz, 1H, H_5), 4.67 (quint, J = 7.3 Hz, 1H, H_7), 4.20 (d, J = 15.9 Hz, 1H, H_6), 4.10 (d, J = 15.9 Hz, 1H, H_6), 3.87 (ddt, J = 16.9, 10.8, 5.6 Hz, 2H, H_3), 3.77 (s, 3H, H_9), 2.83 (sept, J = 7.1 Hz, 1H, H_2), 1.45 (d, J = 7.3 Hz, 3H, H_8), 1.21 (d, J = 7.1 Hz, 3H, H_1), 1.19 (d, J = 7.1 Hz, 3H, H_1).

^{13}C NMR ($CDCl_3$, 100.6 MHz) δ 173.2 (C_D), 167.9 (C_B), 167.8 (C_C), 144.1 (C_E), 143.9 (C_A), 132.3 (C_4), 128.8 (C_{11}), 123.7 (C_F), 119.4 (C_5), 113.6 (C_{10}), 52.6 (C_9), 51.1 (C_3), 48.5 (C_7), 46.2 (C_6), 32.3 (C_2), 20.2 (C_1), 19.9 (C_1), 18.4 (C_8).

IR (ν, cm^{-1}): 2926, 2361, 1744, 1688, 1626, 1602, 1542, 1492, 1454, 1255, 1209.

HRMS: calculated for $C_{20}H_{27}ClN_4O_4$ 422.1721, found 422.1722.

(*E*)-2-(*N*-allyl-2-(2-phenylhydrazono)acetamido)-*N*-cyclohexylbutanamide (3g)

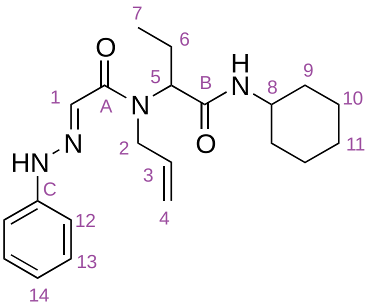

$C_{21}H_{30}N_4O_2$
Mol wt = 370.48 g·mol^{-1}

Compound **3g** was prepared following the general procedure A applied to 2-(2-phenylhydrazono)acetic acid (500 mg, 3.05 mmol), propionaldehyde (220 µL, 3.05 mmol), cyclo-hexylisocyanide (380 µL, 3.05 mmol).

Yield: 58% (653 mg)

R_f : 0.25 (100% Et_2O)

Red solid.

Mp: 95–96 °C

^{1}H NMR ($CDCl_3$, 400 MHz) δ 8.26 (br s, 1H, NH), 7.34 (s, 1H, H_1), 7.32–7.30 (m, 2H, H_{13}), 7.07 (d, *J* = 7.1 Hz, 2H, H_{12}), 7.00–6.97 (m, 1H, H_{14}), 6.40–6.38 (m, 1H, NH), 5.95–5.89 (m, 1H, H_3), 5.23 (d, *J* = 16.9 Hz, 1H, H_4), 5.18 (d, *J* = 10.1 Hz, 1H, H_4), 4.83 (t, *J* = 6.8 Hz, 1H, H_5), 4.34–4.16 (m, 2H, H_2), 3.75–3.76 (m, 1H, H_8), 2.08–2.01 (m, 1H, H_6), 1.91–1.82 (m, 2H, H_9), 1.70–1.67 (m, 3H, $H_{6,}$ H_{10}), 1.62–1.58 (m, 1H, H_{11}), 1.38–1.31 (m, 2H, H_{10}), 1.17–1.12 (m, 3H, H_9, H_{11}), 0.96 (t, *J* = 6.8 Hz, 3H, H_7).

^{13}C NMR ($CDCl_3$, 100.6 MHz) δ 170.1 (C_B), 166.3 (C_A), 143.6 (C_C), 135.3 (C_3), 129.7 (C_{13}), 128.0 (C_1), 122.1 (C_{14}), 117.5 (C_4), 114.0 (C_{12}), 60.2 (C_5), 48.6 (C_8), 47.9 (C_2), 33.3 (C_9), 33.1 (C_9), 25.8 (C_{11}), 25.2 (C_{10}), 22.0 (C_6), 11.2 (C_7).

IR (ν, cm^{-1}): 2931, 2361, 1648, 1538, 1453, 1250.

HRMS: calculated for $C_{21}H_{30}N_4O_2$ 370.2369, found 370.2369

(*E*)-2-(*N*-allyl-2-phenyl-2-(2-phenylhydrazono)acetamido)-*N*-cyclohexyl-4-methylpentanamide (3h)

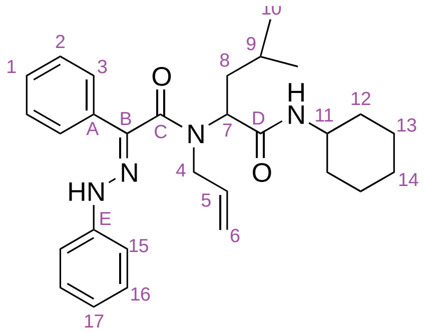

$C_{29}H_{38}N_4O_2$
Mol wt = 474.64 g·mol^{-1}

Compound **3h** was prepared following the general procedure A applied to 2-phenyl-2-(2-phenylhydrazono)acetic acid (673 mg, 2.8 mmol), isovaleraldehyde (300 µL, 2.8 mmol), cyclo-hexylisocyanide (403 µL, 2.8 mmol).

Yield: 52% (660 mg)

R_f: 0.6 (5:5 PE/Et_2O)

Yellow solid

Mp: 67–68 °C

Obtained as a mixture of two rotamers A:B in a 1.2:0.8 ratio

^{1}H NMR ($CDCl_3$, 400 MHz) δ 9.15 (br s, 0.6 H_A, NH), 8.13 (br s, 0.4 H_B, NH), 7.70 (d, J = 7.8 Hz, 0.8H_B, H_3), 7.65 (d, J = 7.8 Hz, 1.2H_A, H_3), 7.44–7.35 (m, 2H, H_2), 7.32–7.24 (m, 3H, 1.2H_A, H_1, H_{15}, H_{16}), 7.16 (d, J = 8.3 Hz, 0.8H_B, H_{15}), 6.94 (t, J = 7.8 Hz, 0.4H_B, H_{17}), 6.90 (t, J = 7.8 Hz, 0.6H_A, H_{17}), 6.05–5.96 (m, 0.4H_B, H_5), 5.96 (br s, 1H, NH), 5.63 (ddt, J = 16.7, 10.3, 6.5 Hz, 0.6H_A, H_5), 5.37 (d, J = 16.7 Hz, 0.4H_B, H_6), 5.25 (d, J = 10.3 Hz, 0.4H_B, H_6), 5.02 (d, J = 16.7 Hz, 0.6H_A, H_6), 4.98 (d, J = 10.3 Hz, 0.6H_A, H_6), 4.93–4.90 (m, 1H, H_7), 4.31 (dd, J = 14.4, 6.5 Hz, 0.4H_B, H_4), 4.04 (dd, J = 14.4, 6.5 Hz, 0.4H_B, H_4), 3.98–3.91 (m, 1H, H_{11}), 3.89–3.77 (m, 1.2H_A, H_4), 2.03–1.94 (m, 2H, H_{12}), 1.74–1.61 (m, 6H, H_8, H_{13}), 1.41–1.36 (m, 2H, H_{14}), 1.25–1.17 (m, 3H, H_9, H_{12}), 1.06 (d, J = 6.0 Hz, 1.8H_A, H_{10}), 1.02 (d, J = 6.0 Hz, 1.8H_A, H_{10}), 0.62 (d, J = 6.0 Hz, 1.2H_B, H_{10}), 0.46 (d, J = 6.0 Hz, 1.2H_B, H_{10}).

^{13}C NMR ($CDCl_3$, 100.6 MHz) δ 169.9 (C_D), 167.9 (A, C_C), 167.7 (B, C_C), 144.7 (A, C_E), 144.3 (B, C_E), 138.1 (A, C_B), 138.1 (B, C_B), 134.3 (A, C_A), 134.0 (B, C_A), 133.5 (A, C_5), 133.2 (B, C_5), 129.6 (B, C_1), 129.4 (B, C_{16}), 129.2 (A, C_{16}), 129.1 (B, C_2), 128.7 (B, C_2), 128.5 (A, C_1), 125.6 (B, C_3), 125.1 (A, C_3), 121.5 (B, C_{17}), 120.5 (A, C_{17}), 119.1 (A, C_6), 118.6 (B, C_6), 113.8 (B, C_{15}), 113.3 (A, C_{15}), 60.2 (B, C_7), 56.6 (A, C_7), 50.2 (C_4), 48.8 (A, C_{11}), 48.6 (B, C_{11}), 37.4 (A, C_8), 37.3 (B, C_8), 33.1 (A, C_{12}), 33.0 (B, C_{12}), 25.5 (B, C_{14}), 25.3 (A, C_{14}), 25.3 (C_9) 24.9 (A, C_{13}), 24.8 (B, C_{13}), 23.2 (A, C_{10}), 22.6 (B, C_{10}), 21.9 (A, C_{10}), 21.4 (B, C_{10}).

IR (v, cm^{-1}): 2932, 2361, 1621, 1540, 1263.

HRMS: calculated for $C_{29}H_{38}N_4O_2$ 474.2994, found 474.2983.

(*E*)-*N*-allyl-*N*-(1-(*ter*-butylamino)-2-methyl-1-oxopropan-2-yl)-2-(2-phenylhydrazono)propanamide (3i)

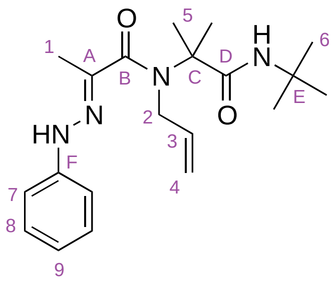

$C_{20}H_{30}N_4O_2$
Mol wt = 358.47 g·mol^{-1}

Compound **3i** was prepared following the general procedure A applied to 2-(2-phenylhydrazono)propanoic acid (500 mg, 2.8 mmol), acetone (210 µL, 2.8 mmol), *tert*-butylisocyanide (317 µL, 1 mmol).

Yield: 50% (505 mg)

R_f: 0.6 (100% Et_2O)

Yellow solid.

Mp: 123–124 °C

^{1}H NMR ($CDCl_3$, 400 MHz) δ 7.32 (br s, 1H, NH), 7.27 (dd, *J* = 8.0, 7.3 Hz, 2H, H_8), 7.07 (d, *J* = 8.0 Hz, 2H, H_7), 6.92 (t, *J* = 7.3 Hz, 1H, H_9), 6.01 (ddt, *J* = 16.9, 10.4, 5.2 Hz, 1H, H_3), 5.73 (br s, 1H, NH), 5.18 (d, *J* = 16.9 Hz, 1H, H_4), 5.17 (d, *J* = 10.3 Hz, 1H, H_4), 4.29 (d, *J* = 5.2 Hz, 2H, H_2), 2.04 (s, 3H, H_1), 1.53 (s, 6H, H_5), 1.31 (s, 9H, H_6).

^{13}C NMR ($CDCl_3$, 100.6 MHz) δ 174.3 (C_D), 170.0 (C_B), 144.4 (C_F), 138.5 (C_A), 137.7 (C_3), 129.7 (C_8), 121.5 (C_9), 117.1 (C_4), 113.8 (C_7), 64.1 (C_E), 51.1 (C_C), 49.1 (C_2), 29.0 (C_6), 24.9 (C_5), 12.7 (C_1).

IR (ν, cm^{-1}): 1684, 1558, 1541, 1508.

HRMS: calculated for $C_{20}H_{30}N_4O_2$ 358.2369, found 358.2370

N-tert-butyl-2-(6a-methyl-3,6-dioxo-2-phenyl-hexahydropyrrolo[3,4-c]pyrazol-5(1H)-yl)acetamide (3a)

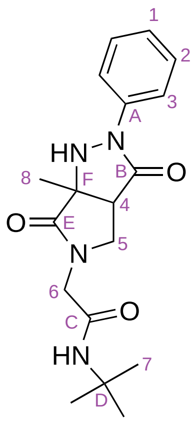

$C_{18}H_{24}N_4O_3$
Mol wt = 344.41 g·mol^{-1}

Compound **3a** was prepared following the general procedure B applied to hydrazone **2a** (100 mg, 0.3 mmol).

Yield: 84% (87 mg)

R_f: 0.3 (AcOEt 100%)

Yellow solid.

Mp: 200 °C (degradation)

^{1}H NMR ($CDCl_3$, 400 MHz) δ 7.83 (d, J = 8.3 Hz, 2H, H_3), 7.37 (dd, J = 8.3, 7.3 Hz, 2H, H_2), 7.17 (t, J = 7.3 Hz, 1H, H_1), 5.52 (br s, 1H, NH *t*Bu), 4.89 (br s, 1H, NH), 3.93 (d, J = 16.2 Hz, 1H, H_6), 3.85 (dd, J = 10.3, 6.3 Hz, 1H, H_5), 3.80 (d, J = 16.2 Hz, 1H, H_6), 3.76 (d, J = 10.3 Hz, 1H, H_5), 3.22 (d, J = 6.3Hz, 1H, H_4), 1.63 (s, 3H, H_8), 1.25 (s, 9H, H_7).

^{13}C NMR ($CDCl_3$, 100.6 MHz) δ 174.6 (C_E), 169.8 (C_B), 166.3 (C_C), 138.1 (C_A), 129.2 (C_2), 125.8 (C_1), 119.5 (C_3), 63.7 (C_F), 52.0 (C_D), 48.0 (C_4, C_6), 47.6 (C_5), 29.0 (C_7), 18.9 (C_8).

IR (ν, cm^{-1}): 1684, 1541, 1497, 1363.

HRMS: calculated for $C_{18}H_{24}N_4O_3$ 344.1848, found 344.1855.

N-tert-butyl-2-(3,6-dioxo-2,6a-diphenyl-hexahydropyrrolo[3,4-c]pyrazol-5(1*H*)-yl)acetamide (3b)

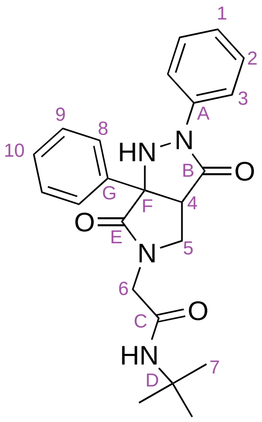

$C_{23}H_{26}N_4O_3$
Mol wt = 406.48 g·mol^{-1}

Compound **3b** was prepared following the general procedure B applied to hydrazone **2b** (100 mg, 0.25 mmol).

Yield: 68% (60 mg)

R_f: 0.7 (AcOEt 100%)

Yellow solid.

Mp: 105–106 °C.

^{1}H NMR ($CDCl_3$, 400 MHz) δ 7.91 (d, *J* = 8.1 Hz, 2H, H_3), 7.66 (d, *J* = 7.6 Hz, 2H, H_8), 7.51–7.45 (m, 3H, H_9, H_{10}), 7.40 (dd, *J* = 8.1, 7.6 Hz, 2H, H_2), 7.20 (t, *J* = 7.6 Hz, 1H, H_1), 5.53 (br s, 1H, NH *t*-Bu), 5.23 (br s, 1H, NH), 3.97–3.84 (m, 4H, H_5, H_6), 3.74–3.72 (m, 1H, H_4), 1.18 (s, 9H, H_7).

^{13}C NMR ($CDCl_3$, 100.6 MHz) δ 173.2 (C_E), 169.0 (C_B), 166.3 (C_C), 138.0 (C_A), 134.9 (C_G), 130.0 (C_{10}), 129.8 (C_9), 129.3 (C_2), 126.4 (C_8), 126.0 (C_1), 119.7 (C_3), 68.9 (C_F), 51.9 (C_D), 48.9 (C_6), 48.2 (C_5), 48.0 (C_4), 28.8 (C_7).

IR (ν, cm^{-1}): 1684, 1558, 1541, 1508, 1457.

HRMS: calculated for $C_{23}H_{26}N_4O_3$ 406.2005, found 406.2004.

_N_-cyclohexyl-2-(6a-methyl-3,6-dioxo-2-phenyl-hexahydropyrrolo[3,4-c]pyrazol-5(1_H_)-yl)acetamide (3c)

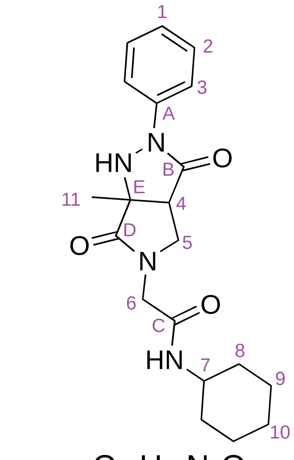

$C_{20}H_{26}N_4O_3$
Mol wt = 370.45 g·mol^{-1}

Compound **3c** was prepared following the general procedure B applied to hydrazone **2c** (100 mg, 0.28 mmol).

Yield: 76% (83 mg)

R_f: 0.2 (AcOEt 100%)

White solid.

Mp: 197–198 °C (degradation)

^{1}H NMR ($CDCl_3$, 400 MHz) δ 7.82 (d, J = 8.1 Hz, 2H, H_3), 7.35 (dd, J = 8.1, 7.5 Hz, 2H, H_2), 7.15 (t, J = 7.5 Hz, 1H, H_1), 5.60 (br s, 1H, NH Cy), 4.88 (br s, 1H, NH), 3.98 (d, J = 15.4 Hz, 1H, H_6), 3.86–3.82 (m, 2H, H_5, H_6) 3.75 (d, J = 10.1 Hz, 1H, H_5), 3.70–3.61 (m, 1H, H_7), 3.22 (d, J = 6.5 Hz, 1H, H_4), 1.79–1.75 (m, 2H, H_8), 1.62 (s, 3H, H_{11}), 1.60–1.49 (m, 4H, H_9, H_{10}), 1.28–1.21 (m, 2H, H_9), 1.02–0.92 (m, 2H, H_8).

^{13}C NMR ($CDCl_3$, 100.6 MHz) δ 174.7 (C_D), 169.9 (C_B), 166.2 (C_C), 138.1 (C_A), 129.3 (C_2), 125.8 (C_1), 119.5 (C_3), 63.8 (C_E), 48.8 (C_4), 48.1 (C_7), 47.7 (C_6), 47.5 (C_5), 33.2 (C_8), 33.1 (C_8), 25.7 (C_{10}), 25.1 (C_9), 18.8 (C_{11}).

IR (ν, cm^{-1}): 1698, 1595, 1541, 1497, 1457, 1362.

HRMS: calculated for $C_{20}H_{26}N_4O_3$ 370.2005, found 370.2002.

N-(4-chlorobenzyl)-2-(6a-methyl-3,6-dioxo-2-phenyl-hexahydropyrrolo[3,4-c]pyrazol-5(1H)-yl)acetamide (3d)

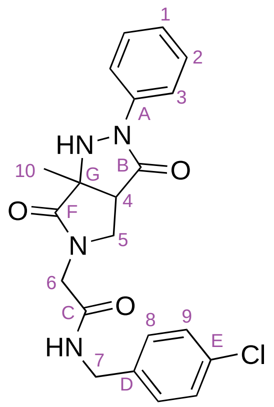

$C_{21}H_{21}ClN_4O_3$
Mol wt = 412.87 g·mol^{-1}

Compound **3d** was prepared following the general procedure B applied to hydrazone **2d** (100 mg, 0.25 mmol).

Yield: 90% (95 mg)

R_f: 0.2 (AcOEt 100%)

White solid.

Mp: 175–176°C (degradation)

^{1}H NMR ($CDCl_3$, 400 MHz) δ 7.78 (d, *J* = 8.3 Hz, 2H, H_3), 7.37 (dd, *J* = 8.3, 7.0 Hz, 2H, H_2), 7.24 (d, *J* = 7.8 Hz, 2H, H_9), 7.19 (t, *J* = 7.0 Hz, 1H, H_1), 7.13 (d, *J* = 7.8 Hz, 2H, H_8), 6.10 (br s, 1H, NH Bn), 4.81 (br s, 1H, NH), 4.41–4.31 (m, 2H, H_7), 4.12 (d, *J* = 15.9 Hz, 1H, H_6), 3.89 (d, *J* = 15.9 Hz, 1H, H_6), 3.89–3.81 (m, 2H, H_5), 3.25 (d, *J* = 5.8 Hz, 1H, H_4), 1.61 (s, 3H, H_{10}).

^{13}C NMR ($CDCl_3$, 100.6 MHz) δ 174.8 (C_F), 169.6 (C_B), 167.2 (C_C), 137.9 (C_A), 136.3 (C_D), 133.7 (C_E), 129.4 (C_8), 129.2 (C_2, C_9), 125.8 (C_1), 119.4 (C_3), 63.6 (C_G), 48.0 (C_4), 47.8 (C_6), 47.3 (C_5), 43.3 (C_7), 18.8 (C_{10}).

IR (ν, cm^{-1}): 1684, 1541, 1276, 1261.

HRMS: calculated for $C_{21}H_{21}ClN_4O_3$ 412.1302, found 412.1290.

N-cyclohexyl-2-(3,6-dioxo-2,6a-diphenyl-hexahydropyrrolo[3,4-c]pyrazol-5(1*H*)-yl)acetamide (3e)

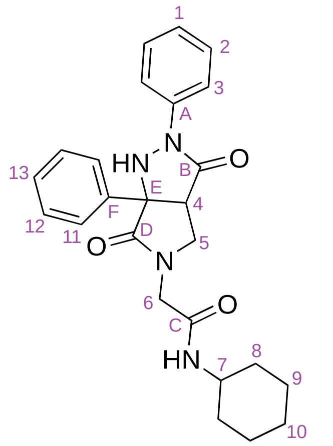

$C_{25}H_{28}N_4O_3$
Mol wt = 432.51 g·mol^{-1}

Compound **3e** was prepared following the general procedure B applied to hydrazone **2e** (100 mg, 0.24 mmol).

Yield: 72% (75 mg)

R_f: 0.7 (AcOEt 100%)

Yellow oil

^{1}H NMR ($CDCl_3$, 400 MHz) δ 7.89 (d, *J* = 8.6 Hz, 2H, H_3), 7.65 (d, *J* = 8.1 Hz, 2H, H_{11}), 7.48–7.42 (m, 3H, H_{12}, H_{13}), 7.39 (dd, *J* = 8.6, 7.8 Hz, 2H, H_2), 7.18 (t, *J*= 7.8 Hz, 1H, H_1), 5.60 (br s, 1H, NH Cy), 5.21 (br s, 1H, NH cycle), 4.01–3.96 (m, 2H, H_6), 3.91–3.87 (m, 2H, H_5), 3.74–3.70 (m, 1H, H_7), 3.66–3.57 (m, 1H, H_4), 1.79–1.48 (m, 4H, H_8, H_9), 1.31–1.16 (m, 2H, H_{10}), 1.00–0.75 (m, 4H, $H_{8,}$ H_9).

^{13}C NMR ($CDCl_3$, 100.6 MHz) δ 173.3 (C_D), 169.0 (C_B), 166.1 (C_C), 138.0 (C_A), 134.7 (C_F), 129.9 (C_{13}), 129.7 (C_{12}), 129.3 (C_2), 126.4 (C_{11}), 125.9 (C_1), 119.7 (C_3), 68.9 (C_E), 48.6 (C_4), 48.3 (C_5), 47.9 (C_7), 33.0 (C_8), 32.9 (C_8), 25.6 (C_{10}), 24.9 (C_9).

IR (ν, cm^{-1}): 1697, 1596, 1542, 1496, 1448, 1363.

HRMS: calculated for $C_{25}H_{28}N_4O_3$ 432.2161, found 432.2169.

Methyl 2-(2-(2-(4-chlorophenyl)-6a-isopropyl-3,6-dioxohexahydropyrrolo[3,4-c]pyrazol-5(1*H*)-yl)acetamido)propanoate (3f)

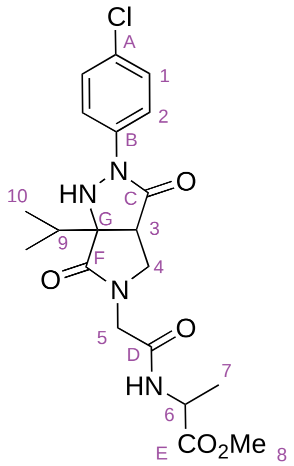

$C_{20}H_{25}ClN_4O_5$
Mol wt = 436.88 g·mol^{-1}

Compound **3f** was prepared following the general procedure B applied to hydrazone **2f** (20 mg, 0.047 mmol).

Yield: 49% (10 mg)

R_f: 0.3 (AcOEt 100%)

Orange oil

Obtained as a mixture of two diastereomers A:B in a 1.1:0.9 ratio :

^{1}H NMR ($CDCl_3$, 400 MHz) δ 7.78 (d, *J* = 8.8 Hz, 1.1H_A, H_2), 7.77 (d, *J* = 8.8 Hz, 0.9H_B, H_2), 7.31 (d, *J* = 8.8 Hz, 2H, H_1), 6.42 (d, *J* = 7.5 Hz, 0.45H_B, NH), 6.41 (d, *J* = 7.5 Hz, 0.55H_A, NH), 5.00 (br s, 0.45H_B, NH), 4.98 (br s, 0.55H_A, NH), 4.54 (q, *J* = 6.8 Hz, 0.55H_A, H_6), 4.52 (q, *J* = 6.8 Hz, 0.45H_B, H_6), 4.28 (d, *J* = 16.4 Hz, 0.45H_B, H_5), 4.23 (d, *J* = 16.4 Hz, 0.55H_A, H_5), 3.89–3.85 (m, 1H, H_4), 3.80–3.72 (m, 2H, H_4, H_5), 3.71 (s, 1.65H_A, H_8), 3.71 (s, 1.35H_B, H_8), 3.28 (d, *J* = 7.5 Hz, 1H, H_3), 2.28 (sept, *J* = 6.5 Hz, 1H, H_9), 1.36 (d, *J* = 6.8 Hz, 1.35H_B, H_7), 1.35 (d, *J* = 6.8 Hz, 1.65H_A, H_7), 1.15–1.09 (m, 6H, H_{10}).

^{13}C NMR ($CDCl_3$, 100.6 MHz) δ 174.2 (C_F), 173.0 (A, C_E), 172.8 (B, C_E), 168.7 (C_C), 166.4 (B, C_D), 166.3 (A, C_D), 136.3 (B, C_A), 136.1 (A, C_A), 130.4 (B, C_B), 130.3 (A, C_B), 128.8 (B, C_1), 128.8 (A, C_1), 120.2 (C_2), 69.0 (B, C_G), 69.0 (A, C_G), 52.6 (C_8), 49.2 (B, C_4), 49.2 (A, C_4), 48.1 (B, C_6), 48.0 (A, C_6), 46.6 (B, C_5), 46.5 (A, C_5), 44.0 (B, C_3), 43.8 (A, C_3), 32.0 (B, C_9), 31.9 (A, C_9), 18.3 (B, C_7), 18.1 (A, C_7), 17.3 (B, C_{10}), 17.2 (A, C_{10}).

IR (ν, cm^{-1}): 2925, 2361, 1681, 1492, 1460, 1360, 1211.

HRMS: calculated for $C_{20}H_{25}ClN_4O_5$ 436,1513, found 436.1515.

(E)-N-cyclohexyl-2-(4-formyl-2-hydroxy-3-(phenyldiazenyl)-1H-pyrrol-1-yl)butanamide (4g)

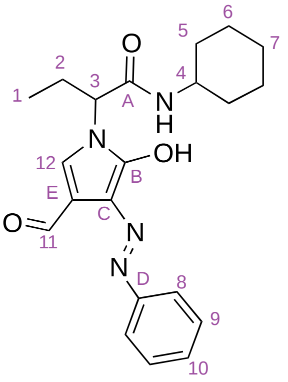

$C_{21}H_{26}N_4O_3$
Mol wt = 382.45 g·mol^{-1}

To a solution of hydrazone **3g** (400 mg, 1.08 mmol) in a 20/80 H_2O/CH_3CO_2H mixture (0.06 M) was added $Cu(OAc)_2$ (215 mg, 1.08 mmol, 1 equiv). The resulting mixture was heated at 80 °C under argon for 3 h. The pH was adjusted to 6 with an aqueous sodium hydrogenocarbonate solution, and the aqueous phase was extracted with AcOEt. Then the organic layers were washed ten times with water (1 mL), dried over anhydrous $MgSO_4$, filtered and concentrated in vacuo. The product was isolated by flash chromatography on silica gel (PE/Et_2O).

Yield: 14% (12 mg)

R_f: 0.4 (Et_2O 100%)

Yellow oil

^{1}H NMR ($CDCl_3$, 400 MHz) δ 9.26 (s, 1H, H_{11}), 8.27 (s, 1H, H_{12}), 7.48 (d, J = 7.8 Hz, 2H, H_8), 7.38 (dd, J = 7.8, 7.3 Hz, 2H, H_9), 7.13 (t, J = 7.3 Hz, 1H, H_{10}), 4.80 (t, J = 7.8 Hz, 1H, H_3), 3.79–3.70 (m, 1H, H_4), 2.15–2.08 (m, 1H, H_2), 1.96–1.82 (m, 3H, H_2, H_5), 1.74–1.66 (m, 3H, H_6, H_7), 1.60–1.50 (m, 1H, H_7), 1.38–1.29 (m, 2H, H_6), 1.20–1.11 (m, 2H, H_5), 0.98 (t, J = 7.3 Hz, 3H, H_1).

^{13}C NMR ($CDCl_3$, 100.6 MHz) δ 184.1 (C_{11}), 168.3 (C_B), 164.6 (C_A), 148.2 (C_{12}), 142.5 (C_D), 129.8 (C_9), 126.3 (C_E), 124.8 (C_{10}), 116.8 (C_8), 114.4 (C_C), 56.8 (C_3), 49.3 (C_4), 33.3 (C_5), 33.1 (C_5), 26.2 (C_2), 25.7 (C_7), 25.2 (C_6), 25.1 (C_6), 10.8 (C_1).

IR (ν, cm^{-1}): 1733, 1717, 1698, 1653, 1558, 1541, 1521, 1508, 1473, 1419, 1276.

HRMS: calculated for $C_{21}H_{26}N_4O_3$ 382.2005, found 382.2008.

5-allyl-2-methyl-6a-phenyl-hexahydropyrrolo[3,4-c]pyrazol-6(6a*H*)-one (6)

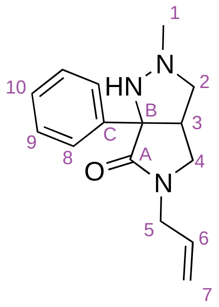

$C_{15}H_{19}N_3O$
Mol wt = 257.33 g·mol^{-1}

A solution of 2-oxo-2-phenylacetic acid (150 mg, 1 mmol), in DCM (0.5 M, 2 mL), with oxalyl chloride (94 µL, 1.1 mmol), with one drop of DMF was stirred for 2 h at room temperature. Then a solution of diallyamine (136 µL, 1.1 mmol) in DCM (1 M, 1.1 mL) and TEA (153 µL, 1.1 mmol) was added dropwise. The solution was filtered and washed with citric acid in water. The aqueous phase was extracted with DCM twice and the combined organic layers were dried over $MgSO_4$, filtered and concentrated in vacuo. The product, used without further purification, was diluted in methanol (1 M, 1 mL) with methylhydrazine (80 µL, 1.5 mmol) and acetic acid (86 µL, 1.5 mmol). The mixture was heated under microwave irradiation at 120 °C (200 W, 13 bar) for 30 min. The mixture was then washed with water and the aqueous phase was extracted with DCM. The organic layers were dried over $MgSO_4$, filtered and concentrated in vacuo.

Yield: 62% (160 mg)

R_f: 0.1 (AcOEt 100%)

Orange oil

^{1}H NMR ($CDCl_3$, 400 MHz) δ 7.40 (d, *J* = 7.1 Hz, 2H, H_8), 7.27 (dd, *J* = 7.8, 7.1 Hz, 2H, H_9), 7.20 (t, *J* = 7.8 Hz, 1H, H_{10}), 5.71–5.61 (m, 1H, H_6), 5.18 (dd, *J* = 15.4, 1.2 Hz, 1H, H_7), 5.14 (dd, *J* = 8.5, 1.2Hz, 1H, H_7), 4.00 (dd, *J* = 15.4, 6.1 Hz, 1H, H_5), 3.81 (dd, *J* = 15.4, 6.1 Hz, 1H, H_5), 3.67 (dd, *J* = 10.6, 7.8 Hz, 1H, H_4), 3.16 (dd, *J* = 10.6, 1.5 Hz, 1H, H_4), 3.04 (dddd, *J* = 8.1, 7.8, 3.6, 1.5 Hz, 1H, H_3), 2.90 (dd, *J* = 10.1, 3.6 Hz, 1H, H_2), 2.73 (dd, *J* = 10.1, 8.1 Hz, 1H, H_2), 2.46 (s, 3H, H_1).

^{13}C NMR ($CDCl_3$, 100.6 MHz) δ 174.2 (C_A), 139.9 (C_C), 131.1 (C_6), 128.7 (C_9), 127.9 (C_{10}), 125.6 (C_8), 118.5 (C_7), 75.7 (C_B), 50.2 (C_4), 45.9 (C_5), 45.9 (C_2), 44.7 (C_3), 44.5 (C_1).

IR (ν, cm^{-1}): 1683, 1491, 1445, 1275.

HRMS: calculated for $C_{15}H_{19}N_3O$ 257.1528, found 257.1531.
